# Differential protein expression in metallothionein protection from depleted uranium-induced nephrotoxicity

**DOI:** 10.1038/srep38942

**Published:** 2016-12-14

**Authors:** Yuhui Hao, Jiawei Huang, Cong Liu, Hong Li, Jing Liu, Yiping Zeng, Zhangyou Yang, Rong Li

**Affiliations:** 1 State Key Laboratory of Trauma, Burns and Combined Injury, Institute of Combined Injury, Chongqing Engineering Research Center for Nanomedicine, College of Preventive Medicine, Third Military Medical University, No. 30 Gaotanyan Street, Shapingba District, Chongqing, 400038, China

## Abstract

The purpose of this study was to investigate the underlying mechanism of metallothionein (MT) protection from depleted uranium (DU) using a proteomics approach to search for a DU toxicity-differential protein. MT−/− and MT+/+ mice were administrated with a single dose of DU (10 mg/kg, i.p.) or equal volume of saline. After 4 days, protein changes in kidney tissues were evaluated using a proteomics approach. A total of 13 differentially expressed proteins were identified using two-dimensional electrophoresis and matrix-assisted laser desorption/ionization time-of-flight mass spectrometry. The validating results showed that the expression of aminoacylase-3 (ACY-3) and the mitochondrial ethylmalonic encephalopathy 1 (ETHE1) decreased significantly after DU exposure; in addition, the reduction in MT−/− mice was more significant than that in MT+/+ mice. The results also showed that exogenous ETHE1 or ACY-3 could increase the survival rate of human embryonic kidney 293 (HEK293) cells after DU exposure. A specific siRNA of ETHE1 significantly increased cell apoptosis rates after DU exposure, whereas exogenous ETHE1 significantly decreased cell apoptosis rates. In summary, ACY-3 and ETHE1 might involve in protection roles of MT. ETHE1 could be a new sensitive molecular target of DU-induced cell apoptosis.

Depleted uranium (DU) is the remaining product after natural uranium is refined and condensed for nuclear fuel-uranium (^235^U). The ^235^U content in DU is quite low, instead, DU is mainly composed of ^238^U. Because of its high penetrability and low price, DU is widely used in counterweights, radiation-protective clothing, and military activities; however, it may cause serious threats to health^1^,^2^. DU is a heavy metal and can emit α and β particles; thus, it exhibits both heavy metal toxicity and radiotoxicity. The kidney is the major target organ of acute DU exposure, and DU can cause severe necrosis of renal proximal tubule endothelial cells, ultimately leading to acute renal failure or even death^3^,^4^,^5^. The following mechanisms of action underlie the nephrotoxicity of DU^6^: (1) altering ion transport in cells to inhibit the oxidative phosphorylation of mitochondria and utilization of adenosine triphosphate (ATP); (2) altering the expression of certain genes (associated with inflammation, oxidative stress, and homeostasis); and (3) increasing oxidative stress levels and decreasing anti-oxidation in cells, thus increasing DNA damage and promoting apoptosis. However, the molecular mechanisms underlying DU-induced nephrotoxicity are more complex, requiring in-depth studies to identify valuable sensitive molecular targets. The biological effect of low-dose DU chronic exposure may be different from acute DU exposure. Chronic uranium exposure induced increasing glutathione in rats without any nephrotoxicity^7^. Vicente Vicente *et al*. reported^8^ that, chronic uranium exposure could decrease the aminoglycoside toxicity threshold and predispose to acute renal failure.

For the treatment of DU intoxication, scholars mainly focused on DU decorporation in the body using synthetic chelating agents, and a series of agents have been developed^9^,^10^,^11^,^12^,^13^. However, there are few drugs with low toxicity that are highly effective in promoting the excretion of DU. Catechol-3,6-bis(methyleiminodiacetic acid) (CBMIDA) has been shown to reduce uranium burden without causing renal damage, but it also has low gastrointestinal absorption and the effect related to pH^14^.

Metallothionein (MT) is a family of low molecular weight, sulphur-containing proteins and plays an important role in metal metabolism and protects cells against the toxic effects of radiation, alkylating agents and oxygen free radicals^15^. Our previous study^16^ showed that pre-treatment with zinc results in significant detoxification effects in rats with acute DU intoxication. The detoxification mechanism might be associated with the synthesis of MT and enhanced anti-oxidative functions. Further study^17^ showed that MT-knockout mice (MT−/−) display significantly higher serum creatinine and urea nitrogen, severe pathological injury, a significant reduction of the activity of anti-superoxide anion free radicals, catalase, glutathione peroxidase, and superoxide dismutase, and increased levels of malondialdehyde after DU exposure compared with wild-type mice (MT+/+). These results suggested that MT might play critical roles in antagonizing DU-induced nephrotoxicity, leading to new perspectives regarding the prevention and treatment of DU-induced injury. Furthermore, Jiang *et al*. reported^18^ that MT exhibits significant protective functions in nematodes after DU exposure.

To further study the molecular mechanism underlying the resistance to DU-induced nephrotoxicity conferred by MT and investigate the effects of DU exposure on the expression of proteins in kidney tissues, in the present study, we first performed two-dimensional electrophoresis (2-DE) and stained proteins with a silver stain which exhibits higher sensitivity. The obtained gel images were analysed using Image Master 2D Elite 3.01. Then, 2-DE was performed again, and the proteins were stained with Coomassie Brilliant Blue. The differentially expressed protein spots visible to the naked eye were selected for matrix-assisted laser desorption/ionization time-of-flight mass spectrometry (MALDI-TOF-MS) to perform peptide mass fingerprinting (PMF) analysis, and the PMF results were subjected to searches using the Mascot database. The differences in protein expression in kidney tissues following DU exposure in MT−/− mice and MT+/+ mice were analysed to investigate the mechanism of action underlying DU-induced nephrotoxicity and the protective function of MT, to obtain many clues regarding DU intoxication and the protection mechanism of MT.

## Results

### Analysis of gel images following 2-DE

**Figure 1:**
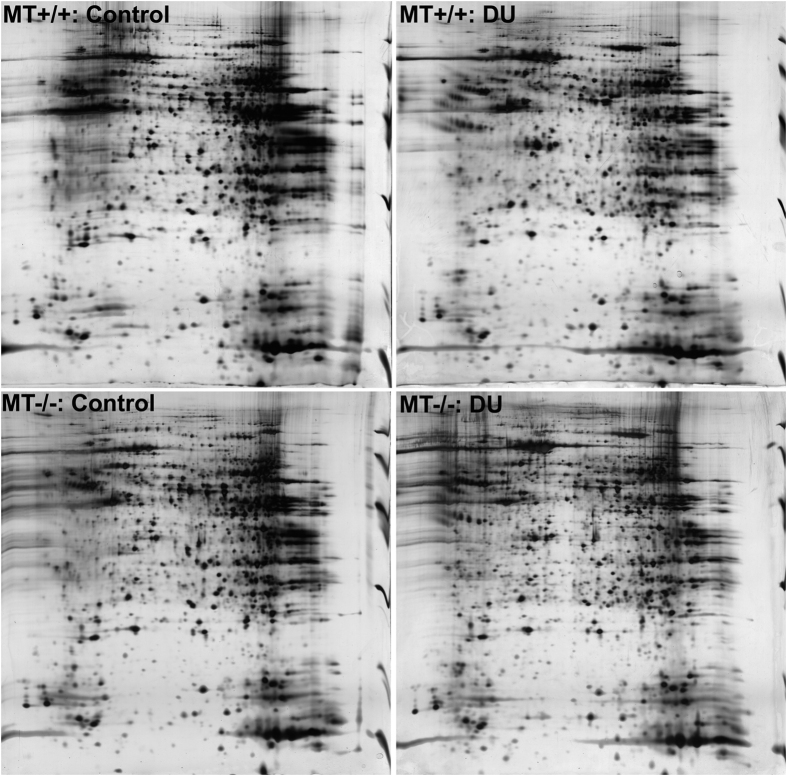
Mice kidney protein separation. MT−/− and MT+/+ mice were administrated with a single dose of DU (10 mg/kg, i.p.) or equal volume of saline (control). After 4 days, kidney proteins were separated by 2-DE (first dimension, 18-cm 3–10NL IPG strips, second dimension, 12% SDS-PAGE) and visualised by silver staining.

**Figure 2:**
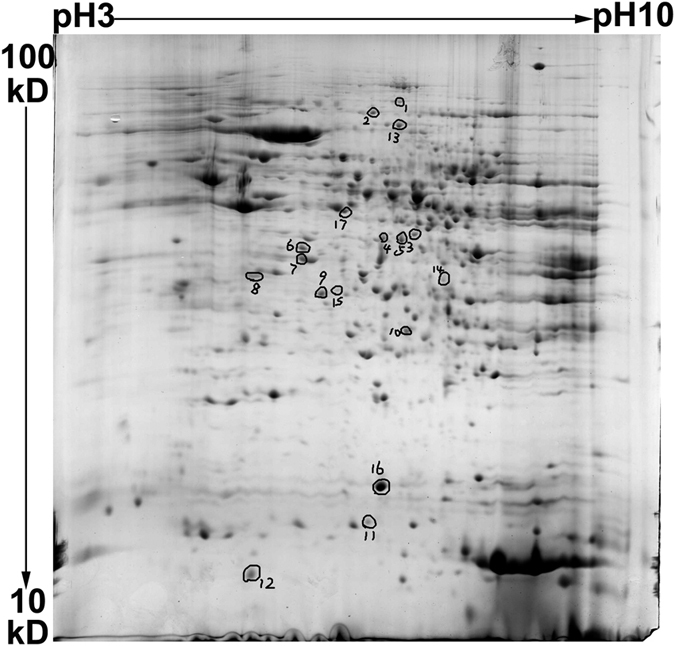
Representative 2-DE (the control sample of the first set of gel images) showing spot numbers of identified proteins. Corresponding identifications, made by PMF are reported in Table 1.

To investigate the molecular mechanism underlying the enhanced DU sensitivity observed after MT knockout, the differences in protein samples from kidney tissues collected after exposure of MT+/+ mice and MT−/− mice to DU or normal saline for 4 d were evaluated using 2-DE. Typical results of silver staining of 2-DE images of samples in all groups are shown in Fig. 1. Image analysis was conducted using Image Master 2D Elite 3.01. A total of 5,488 protein spots (about 1,300/gel) were discovered in these 4 groups, and the matching rate between two groups reached 78.042–80.88%. Pairwise comparisons between these 4 groups revealed 43 differential points using a 2-fold difference as a cut-off. After the images stained with silver were analysed using ImageMaster 2D Elite 3.01, 17 differentially expressed protein spots visible to the naked eye on Coomassie Brilliant Blue-stained gels were selected. The specific locations are shown in Fig. 2.

### Mass spectrometry analysis of differentially expressed proteins

In-gel enzyme digestion was performed on 17 differentially expressed protein spots visible to the naked eye on Coomassie Brilliant Blue-stained gels. PMF analysis was performed using MALDI-TOF-MS. The results identified using PMF were subjected to database searches. The search results with a Mascot search score higher than 64 were selected, and a total of 13 differentially expressed proteins were identified.

Table 1 provides detailed results of the mass spectrometry identification of these 13 differentially expressed proteins. According to the changing trends in each group (Table 2), these proteins were classified into 7 groups. (1) Under normal physiological conditions, the protein expression levels of ezrin, transthyretin, and ATP synthase-coupling factor 6 in MT−/− mice were higher than in MT+/+ mice. (2) Under physiological conditions, the protein expression levels of phosphotriesterase-related protein, λ-crystallin homolog, fructose-1,6-bisphosphatase 1, propionyl-CoA carboxylase α chain, and glycerol-3-phosphate dehydrogenase in MT−/− mice were lower than in MT+/+ mice. (3) Under physiological conditions, the protein expression levels of sarcosine dehydrogenase, N(G), N(G)-dimethylarginine dimethylaminohydrolase 1, aminoacylase-3 (ACY-3), ethylmalonic encephalopathy 1 (ETHE1) and superoxide dismutase (SOD) between MT−/− and MT+/+ mice did not exhibit differences. (4) The proteins that showed increased expression after the addition of DU and whose expression significantly increased in MT+/+ mice included λ-crystallin homolog and ATP synthase-coupling factor 6. (5) The one protein that showed increased expression after the addition of DU and did not exhibit a difference in the increase observed in these two types of mice was transthyretin. (6) The proteins that showed decreased expression after the addition of DU and whose expression was significantly decreased in MT−/− mice included SOD, ETHE1, and ACY-3. (7) The proteins that presented decreased expression after the addition of DU and did not exhibit a difference in the decrease observed in these two types of mice included sarcosine dehydrogenase, N(G), N(G)-dimethylarginine dimethylaminohydrolase 1, ezrin, phosphotriesterase–related protein, fructose-1,6-bisphosphatase 1, propionyl-CoA carboxylase α chain, and glycerol-3-phosphate dehydrogenase.

### Validation of the key differentially expressed proteins, ETHE1 and ACY-3, and their protective effects on cell survival rates after DU exposure

**Figure 3:**
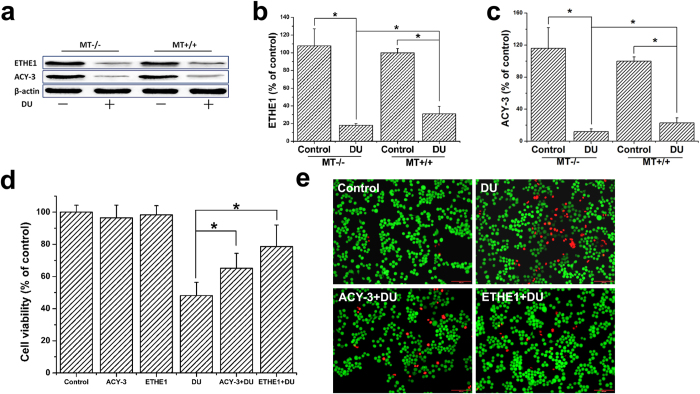
After DU exposure, the expressions of ETHE1 and ACY-3 in MT−/− and MT+/+ mice and the effects of exogenous ETHE1 and ACY-3 on HEK293 cell survival rates. MT−/− and MT+/+ mice were administrated with a single dose of DU (10 mg/kg, i.p.) or equal volume of saline (control). After 4 days, ETHE1 and ACY-3 proteins in kidney were analysed by western blotting (**a**). Quantitative analyses of western blots of ETHE1 (**b**) and ACY-3 (**c**). In addition, HEK293 cells were pretreated with or without ETHE1 (100 ng/ml) or ACY-3 (100 ng/ml) for 0.5 h and then incubated in the absence or presence of DU (500 μM) for 24 h. The cell viability was measured by a CCK-8 assay (**d**), and Calcein-AM/PI staining (**e**) was used for analysis of the living cell (yellow-green fluorescence) and dead cell levels (red fluorescence) as described in Materials and Methods. Results represent means ± SD of five separate experiments. The indicators were evaluated by a two-way ANOVA for two independent variables and by a one-way ANOVA for one variable. *p < 0.05, LSD test for multiple comparisons.

Proteins of interest among the 13 identified differentially expressed proteins were selected for quantitative protein detection. The levels of ACY-3 and ETHE1 in the kidney tissues of MT−/− and MT+/+ mice both decreased after DU injection, and the reduction in MT−/− mice was even more significant (Fig. 3a,b and c). These results were consistent with the results of 2-DE. The relevant findings for SOD have been confirmed in previous study^15^, and the SOD levels in MT−/− mice significantly decreased compared to those in MT+/+ mice after DU exposure.

In addition, the results showed that pretreatment with exogenous ETHE1 or ACY-3 protein could increase the survival rate of HEK293 cells after DU exposure, and the effect of exogenous ETHE1 seemed to be more significant (Fig. 3d). Calcein-AM/PI staining also showed that exogenous ETHE1 or ACY-3 protein could reduce the number of dead cells induced by DU (Fig. 3e). These findings indicated that ACY-3 and ETHE1 might involve in protection roles of MT.

### Relationship between DU exposure and the expression of the ETHE1 protein, and role of ETHE1 on cell survival rates after DU exposure

**Figure 4:**
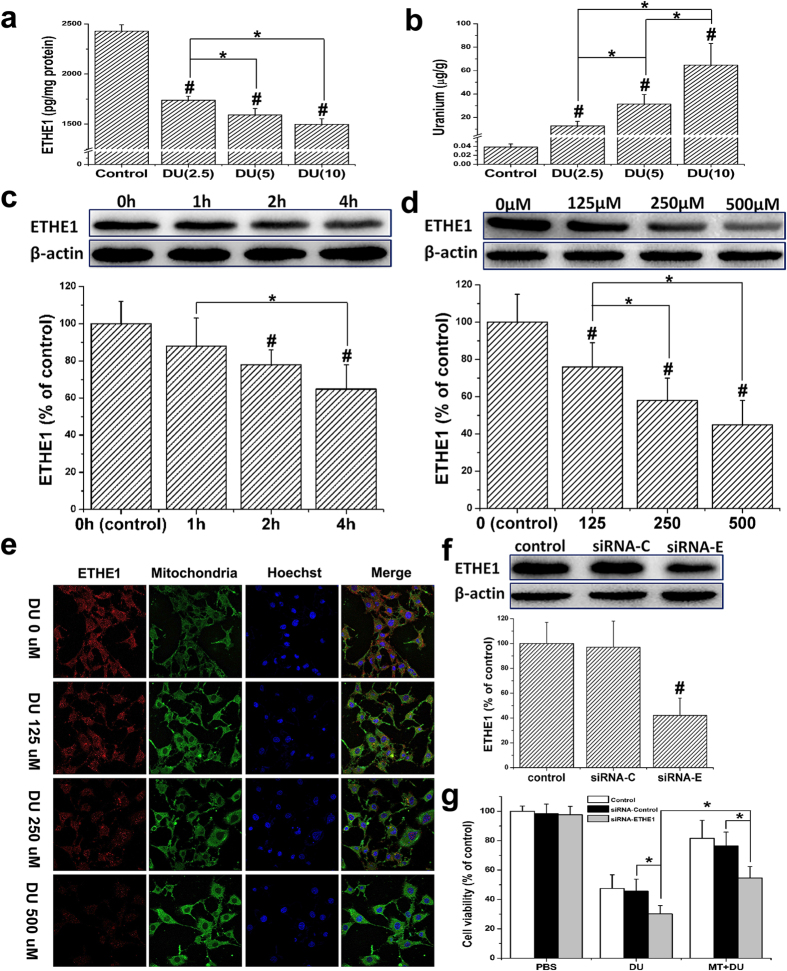
Effect of DU exposure on the expression of the ETHE1 protein, and role of a specific siRNA of ETHE1 on cell survival rates after DU exposure. MT+/+ mice were administrated with DU at a dose of 0, 2.5, 5, or 10 mg/kg. After 4 days, the ETHE1 levels in kidney tissues (**a**) were detected by ELISA and the content of uranium (**b**) was measured by inductively coupled plasma mass spectrometry (ICP-MS). HEK293 cells were treated with DU (500 μM) for different time (0~4 h) (**c**) or different concentrations of DU (0~500 μM) for 4 h (**d**), respectively, and then analysed ETHE1 by Western blotting. (**e**) HEK293 cells were treated with different concentrations of DU (0~500 μM) for 24 h. Then the cells were fixed and probed with ETHE1 antibody to detect subcellular localization of ETHE1 (red), nuclei were visualized by staining with Hoechst (blue), and mitochondria were visualized by staining with Mito-Tracker Green (green). Intracellular localization of ETHE1 was visualized by confocal microscopy. (**f**) Representative western blots showed the levels of ETHE1 in HEK293 cells treated with control siRNA (100 nM) or ETHE1 siRNA (100 nM). siRNA-C, siRNA-control; siRNA-E, siRNA-ETHE1. (**g**) After being transfected with ETHE1-specific siRNA or control siRNA, cells were pretreated with or without MT (100 nM) for 24 h and then incubated in the absence or presence of DU (500 μM) for 24 h. Cell viability was determined by a CCK-8 assay. Results represent means ± SD of five separate experiments. ^#^*p *< 0.05, compared with control group; *p < 0.05, with a one-way ANOVA and LSD test for multiple comparisons.

After a single peritoneal injection of DU into mice at a dose of 0, 2.5, 5, or 10 mg/kg, the ETHE1 levels detected in kidney tissues after 4 days gradually decreased with an increase in the exposure dose and uranium accumulation (Fig. 4a and b), suggesting that ETHE1 might be a DU toxicity-sensitive protein.

The conclusion based on animal experiments was further confirmed in HEK293 cell experiments. ETHE1 levels changed in a dose- and time-dependent manner after DU exposure (Fig. 4c and d). In addition, we found ETHE1 co-localized with the mitochondria in cells, which confirmed that ETHE1 mainly localized to the mitochondria and was a DU toxicity-sensitive protein (Fig. 4e). To clarify the function of ETHE1 in the nephrotoxicity of DU, we obtained an HEK293 cell line with a low level of ETHE1 expression through siRNA interference. The western blot results confirmed that the expression of ETHE1 protein decreased after siRNA interference (Fig. 4f). Furthermore, the reduction of the survival rate of cells after DU exposure was more significant compared with that in negative control cells (Fig. 4g). Application of exogenous MT could still significantly increase the survival rate of cells after DU exposure, and the survival rate of siRNA-ETHE1 cells was lower than that of negative control cells (transfected with a blank vector), suggesting that ETHE1 might be involved in the process of DU-induced nephrotoxicity. In addition, the protection of DU by MT might be partially achieved through the action of ETHE1.

### Effects of ETHE1 on cell apoptosis after DU exposure

**Figure 5:**
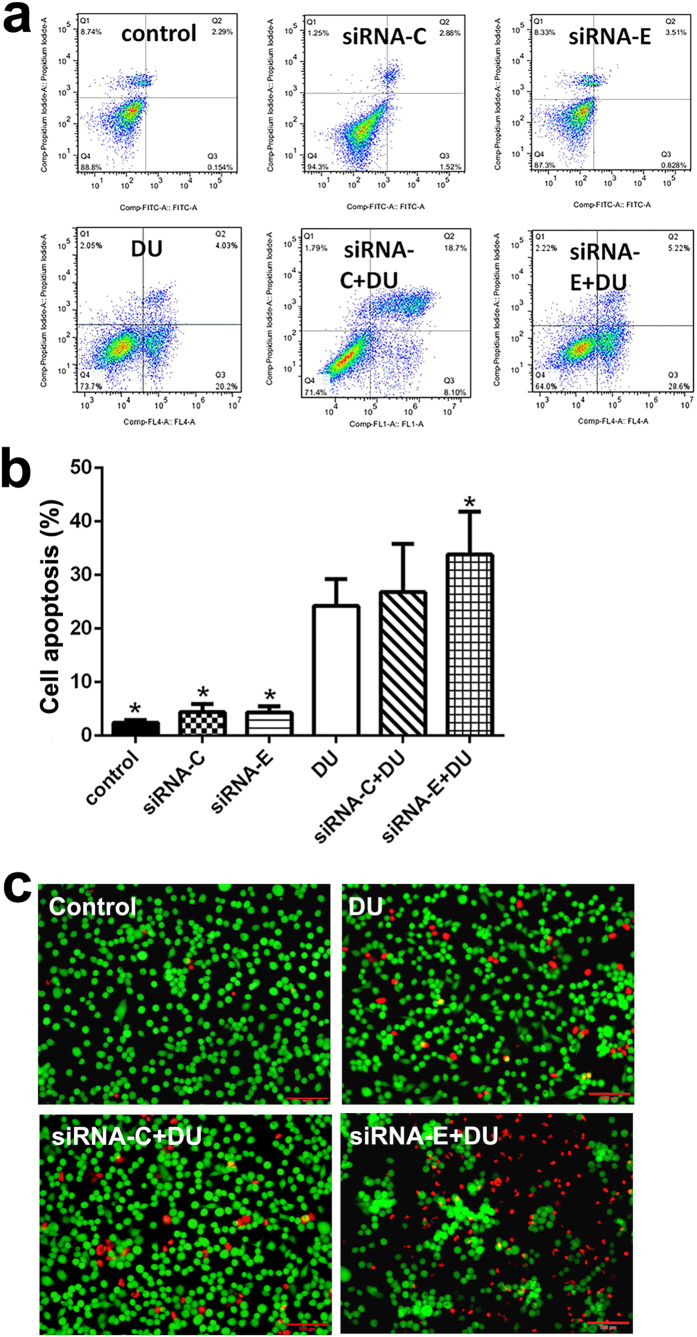
Effect of a specific siRNA of ETHE1 on cell apoptosis after DU exposure. After being transfected with ETHE1-specific siRNA (100 nM) or control siRNA (100 nM), HEK293 cells were incubated in the absence or presence of DU (500 μM) for 24 h. siRNA-C, siRNA-control; siRNA-E, siRNA-ETHE1. (**a**) Apoptosis was measured by flow cytometry, followed by Annexin V-FITC (FL 1 channels) and PI (FL 2 channels) double staining. (**b**) The percentage of apoptosis was counted including early apoptosis (in the lower-right quadrants) and late apoptosis (in the upper-right quadrant). (**c**) Calcein-AM/PI staining was used for analysis of the living cell (yellow-green fluorescence) and dead cell levels (red fluorescence) as described in Materials and Methods. Results represent means ± SD of five separate experiments. *p < 0.05, compared with DU-treated cells.

Next, our findings showed that the cell apoptosis rate in HEK293 cells subjected to ETHE1 siRNA interference was higher than that in negative control cells after DU exposure (Fig. 5a and b). Calcein-AM/PI staining further verified that DU induced more dead cells in siRNA-ETHE1 cells than in negative control cells (Fig. 5c).

**Figure 6:**
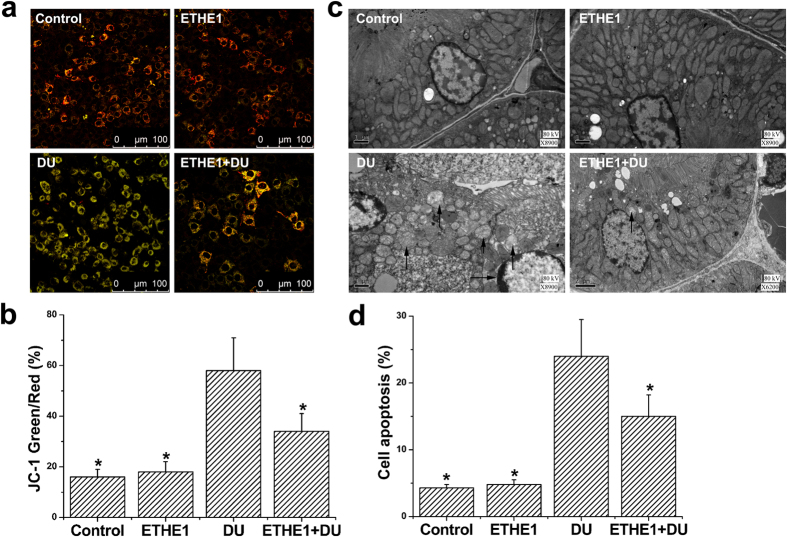
Effect of exogenous ETHE1 protein on the mitochondrial membrane potential and cell apoptosis after DU exposure. HEK293 cells were pretreated with or without ETHE1 (100 ng/ml) for 0.5 h and then incubated in the absence or presence of DU (500 μM) for 24 h. (**a**) The cells were stained with JC-1 probe and visualized by confocal microscope. (**b**) Mitochondrial depolarization was expressed as the ratio of green to red fluorescence. (**c**) The ultrastructures were observed by transmission electron microscopy. (↑) swollen mitochondria; (→) nuclear chromatin concentration and edge accumulation. (**d**) Apoptosis was analysed by flow cytometry, followed by Annexin V-FITC (FL 1 channels) and PI (FL 2 channels) double staining. Results represent means ± SD of five separate experiments. *p < 0.05, compared with DU-treated cells.

A previous study by our group showed that DU exposure could activate the mitochondrial-dependent apoptosis pathway^19^. This study showed that pretreatment with exogenous ETHE1 protein could significantly relieve the DU-induced reduction of mitochondrial membrane potential (Fig. 6a and b) and reduce cell apoptosis (Fig. 6d). The ultrastructures appeared to be normal under TEM pretreatment with ETHE1 (100 ng/ml) alone, and swollen mitochondria and nuclear chromatin concentration and edge accumulation were seen after DU exposure (Fig. 6c). Large numbers of apoptotic cells were also observed after DU exposure. However, pretreatment with exogenous ETHE1 protein, apoptotic cells and the severity of mitochondria damage were significantly lower. Therefore, ETHE1 might be involved in DU-induced cell apoptosis.

## Discussion

DU is employed extensively in civil and military activities. The kidney is the target organ of DU intoxication. Investigation of the mechanism underlying DU intoxication and the search for effective preventive measures deserve considerable attention worldwide. Previous studies^16^,^17^,^19^ by our group showed that MT might have an important preventive function in DU-induced nephrotoxicity. The proteomics approach applied in this study identified a total 13 differentially expressed proteins based on a greater than 2-fold difference in expression and a Mascot search score higher than 64. These proteins included ezrin, ACY-3, ETHE1, transthyretin, ATP synthase-coupling factor 6, phosphotriesterase–related protein, λ-crystallin homolog, fructose-1,6-bisphosphatase 1, propionyl-CoA carboxylase α chain, glycerol-3-phosphate dehydrogenase, sarcosine dehydrogenase, N(G), N(G)-dimethylarginine dimethylaminohydrolase 1, and SOD. The functions of these proteins are mainly related to sugar and fatty acid metabolism, electron transport in the respiratory chain, and the anti-oxidative defence system. These results provide new clues for exploration of MT protection from DU.

MT is metal-binding protein involved in diverse processes, including metal homeostasis and detoxification, the oxidative stress response and cell proliferation. Aberrant expression and silencing of these genes are important in a number of diseases^20^. Our previous study^16^,^17^ showed that MT might play an important role in antagonizing DU-induced nephrotoxicity, however, Ecker *et al*. showed^21^ that, yeast metallothionein conferred resistance to copper and cadmium, but not to uranium (data not showed). We think the difference may be related to species, exposure time and concentration. Michon *et al*. reported^22^ that, 5 moles of U (VI) could be complexed by 1 mole of MT. In fact, though MT is the metalloprotein with low binding site density, MT could significantly alleviate depleted uranium-induced nephrotoxicity. In addition, *Caenorhabditis elegans* MT-1 appeared to be important for DU accumulation^18^,^23^.

Under normal conditions, the kidney tissues of MT−/− and MT+/+ mice exhibit differences in sugar and lipid metabolism and ATP synthesis. Similar to the results of previous study^24^, enzymes detected in heart tissues that are involved in lipid metabolism showed differences in MT−/− and MT+/+ mice. A genomics study by Miura *et al*. revealed^25^ 41 differentially expressed genes between the kidneys of MT−/− and MT+/+ mice, 11 of which were associated with energy metabolism.

After DU exposure, it was most notable that proteins including SOD, ETHE1, and ACY-3 showed decrease in expression, and the reduction in their expression was more significant in MT−/− mice. SOD, also known as liver protein or orgotein, is an important anti-oxidant enzyme in organisms. It is extensively distributed in a variety of organisms including animals, plants, and microorganisms. SOD can remove harmful substances produced during metabolism in organisms, defend and block cell damage caused by oxygen free radicals, and repair damaged cells over time. The pressures of modern life, environmental contamination, a variety of types of radiation, and excessive exercise all cause the production of large amounts of oxygen free radicals; therefore, the status of SOD in the biological antioxidant mechanism is becoming increasingly important. The results of this study suggest that one important mechanism of DU intoxication is to induce a reduction of SOD to increase the production of reactive oxygen species in the body, which further confirm the results of a previous study^17^. On the other hand, these results also suggest that the anti-DU intoxication function of MT is associated with the reduction of SOD levels in kidney tissues.

There are few available studies on ACY-3. It has been reported that ACY-3 mainly localizes to the cytoplasm of proximal tubule epithelial cells, participates in the detoxification process of foreign substances in kidney, and is associated with nephrotoxicity induced by metabolic products (mercapturic acid)^26^. Many toxic substances can be excreted from the body through the mercapturic acid metabolic pathway^27^,^28^. The mercapturic acid metabolic pathway plays an important role in the protection of kidney functions^29^. Masereeuw *et al*. reported^30^ that in the renal proximal tubule brush border, multidrug resistance-associated protein 2 (MRP2) is responsible for the secretion of mercapturic acid into the urine. ACY-3 can catalyse the deacetylation of mercapturic acid, and the product of this reaction is non-toxic. However, with the action of cysteine-S-conjugated β-lyase, toxic substances are produced in the kidney. The precise enzymatic reaction in this metabolic process is still not clear. Mouse ACY-3 is composed of 4 homologous monomers. Each monomer is approximately 35 kDa. ACY-3 localizes to the apical membrane of epithelial cells in the S1 segment of the renal proximal tubule. The resistance of ACY-3 to toxic substances in the kidney, thereby achieving protection of mitochondria in the S1 segment, can be explained by the following mechanisms: (1) reduction of the chance of activation of cysteine-S-conjugates (the production of toxic substances in the kidney is catalysed by mitochondrial cysteine-S-conjugated β-lyase); and (2) possible maintenance of the mercapturic acid gradient in cells and lumens through interaction with other proteins to promote the excretion of mercapturic acid by MRP2^26^. The results of this study indicated that ACY-3 was present in the kidney tissues of both MT−/− and MT+/+ mice and that the level of ACY-3 in the kidney tissues of MT−/− mice was slightly higher than in MT+/+ mice. Upon DU exposure, ACY-3 levels decreased significantly in the kidney tissues of both MT−/− and MT+/+ mice. The reduction of ACY-3 expression in MT−/− mice was even more significant, with ACY-3 expression being nearly undetectable in these mice. These results suggested that after DU intoxication, the level of ACY-3 in kidney tissues changed significantly; thus, the kidney was more prone to injury. MT could reduce this injury to some extent. Due to the loss of the MT gene, MT−/− mice exhibited significantly decrease in ACY-3 expression levels; therefore, ACY-3 might not exert a protective function on DU-induced mitochondrial injury and apoptosis. Thus, MT−/− mice were more sensitive to DU-induced nephrotoxicity. Further in-depth studies are still required to understand the roles of ACY-3 in DU intoxication and protection roles by MT.

ETHE1 is a mitochondrial sulfur dioxygenase discovered in recent years. ETHE1 exhibits a molecular weight of 30 kDa and is associated with energy production. The protein is homodimeric and contains a metallo-β-lactamase domain. ETHE1 is involved in metabolic homeostasis in mitochondria and can bind to nuclear transcription factors and translocate to the cytoplasm to inhibit p53-induced apoptosis^31^. Studies^32^,^33^,^34^ have shown that a defect in the ETHE1 gene can induce a severe mitochondrial disease (ethylmalonic encephalopathy), causing energy metabolism disorders in cells. The results of this study showed that ETHE1 levels in the kidney tissues of MT−/− and MT+/+ mice decreased significantly after DU exposure, suggesting the possibility of increased cell apoptosis. The reduction of ETHE1 expression observed in MT−/− mice was even more significant. In addition, exogenous MT could significantly increase the survival of HEK 293 cells after DU exposure, and the survival rate of ETHE1 siRNA-treated cells was lower than that of the negative control cells (transfected with empty vectors). These results further indicated that ETHE1 is involved in the DU-induced nephrotoxicity process. MT might reduce DU-induced kidney cell injury partially through inhibiting the reduction of ETHE1. The results of this study also suggested that DU exerted its cytotoxicity function and promoted kidney cell apoptosis through the reduction of ETHE1, which might represent a new route of DU intoxication. Further analyses confirmed that ETHE1 mainly localized in the mitochondria and that following DU exposure, HEK293 cells subjected to ETHE1 knockdown through siRNA interference exhibited higher apoptosis rates compared with the negative control cells. Exogenous ETHE1 protein could significantly relieve the reduction of the mitochondrial membrane potential, alleviated the swelling of mitochondria under TEM and reduce cell apoptosis after DU exposure, suggesting that ETHE1 might be involved in DU-induced cell apoptosis and could be a new DU toxicity-sensitive protein.

In summary, the levels of SOD, ETHE1, and ACY-3 significantly decreased after DU exposure, and this phenomenon was more prominent in MT−/− mice. These results suggested that these proteins might be involved in the process of DU-induced nephrotoxicity, and MT might reduce kidney injury to some extent through altering the concentrations of these proteins. Furthermore, ETHE1 could represent a new DU toxicity-sensitive protein and is associated with DU-induced cell apoptosis. This study provides new targets for the prevention and treatment of DU toxicity.

## Materials and Methods

### Animals and treatments

MT-null mice, which were deficient in MT-1 and MT-2 genes, and homozygous wild-type mice were obtained from the Murdoch Institute of the Royal Children’s Hospital (Parkville, Australia) and bred as previously reported^15^. Male mice aged 6–8 weeks with an approximately equal mean body weight were selected for experiment. All experimental procedures were approved by the Animal Care Committee of Third Military Medical University (Chongqing, China) and were in strict accordance with “Principles of laboratory animal care” (NIH publication No. 85-23, revised 1985) regarding the care of animals used for experimental purposes.

Both MT+/+ and MT−/− mice were randomly divided into two groups (control and DU), each consisting of 10 animals (5 animals for 2-DE experiments and 5 animals for western blot analysis), and treated as follows: mice were administrated with a single dose of DU (10 mg/kg, i.p.) or equal volume of saline. The source and constituent of DU was the same as that in the previous study^35^, with 99.75% ^238^U, 0.20% ^235^U and trace ^234^U. The animals were sacrificed 4 days after DU or saline injection. Kidney samples were collected and analysed for 2-DE and western blot, respectively.

### 2-DE and image analysis

Protein 2-DE was performed as previously described^36^,^37^. Frozen kidney tissues were ground into powder under low-temperature conditions. Protein lysis buffer was added at a 5 × volume, and tissues were homogenized in an ice bath for 1 min using a homogenizer. The resultant homogenates were ultra-sonicated 3 times using 5–8 bursts each time. The samples were placed at room temperature for 10 min and then centrifuged at 12,000 g for 20 min at 4 °C. The supernatant was collected and stored at −80 °C for future use. Protein concentrations were determined using bicinchoninic acid (Beyotime, Haimen, Jiangsu, China) colorimetry.

Two-dimensional electrophoresis through isoelectric focusing (IEF) in the first dimension with an immobilized pH gradient (IPG) was performed as follows. A specific amount of protein was accurately aspirated (150 μg of protein loaded for silver-stained samples and 1.0 mg for Coomassie Brilliant Blue-stained samples), and rehydration buffer was added to a final volume of 350 μL. After thorough mixing, the samples were loaded into the IPG gel wells between the positive and negative electrodes. The protective membrane of the pre-made IPG gel strip (18 cm, pH 3~10, NL) (BioRad, Hercules, CA, USA) was then removed, and the samples in the gel well were covered with the strip, with the gel side facing downward. The gel strip was slowly pressed down to avoid bubbles. The top of the gel strip was then evenly sealed with 1 ml of cover oil. The cover was secured, and IEF was performed in an IPG-IEF apparatus (BioRad). After the IEF step was completed, the IPG gel strip was balanced. Sodium dodecyl sulfate polyacrylamide gel electrophoresis (SDS-PAGE, 12%), as the second dimension in 2-DE, was then performed vertically on the PH-balanced IPG gel strip. The settings of the electrophoresis parameters were as follows: 20 mA/gel and electrophoresis at a constant current for 40 min, followed by 30 mA/gel electrophoresis at a constant current until bromophenol blue reached the end of the gel. The gel was then stained with silver nitrate or Coomassie Brilliant Blue (BioRad).

The stained electrophoresis gels were scanned in an optical image scanner (BioRad) in 256 colours at a resolution of 300 dpi. Image analysis was performed using Image Master 2D Elite 3.01 software (BioRad) to obtain the isoelectric point and relative concentration of each protein spot.

### PMF analysis of differentially expressed proteins and preliminary identification of proteins through database searches

Protein spot digestion and protein identification were performed as previously described^38^. The target protein spots in the 2-DE gels were selected and submitted to trypsin digestion using the Montage In-Gel DigestZP Kit (Millipore, Bedford, MA, USA). A Bruker REFLEX III MALDI-TOF-MS instrument (Bruker Daltonics, Bremen, Germany) was used for the analysis. The mass accuracy of the mass spectral peak was calibrated using the auto-digestion peak of trypsin. The mass spectrometry data were subjected to NCBI (version 18/09/2010) database searches using Mascot software (version 2.2.04; Matrix Science, London, UK). According to the input protein isoelectric point, molecular weight range, and PMF data, searches for proteins with matching parameters were performed in the database. The following procedure for database searching is provided at http://www.matrixscience.com. MASCOT search parameters were as follows: no molecular weight restriction, 2 miscleavages allowed, enzyme = trypsin, variable modifications = oxidation (M)/carbamidomethyl (C), peptide tolerance = 0.3 Da, peptide charge = 1+. In the whole experiment, masks and gloves were worn to reduce keratin contamination.

### Enzyme-linked immunosorbent assay (ELISA)

Another 20 mice (only MT+/+) were administrated with DU at a dose of 0, 2.5, 5, or 10 mg/kg. The fourth day post DU injection, kidney tissues were lysed, and the total protein content was determined. The level of ETHE1 in the kidney tissue was measured by ELISA kit (CUSABIO, Wuhan, Hubei, China). The absorbance was measured at 450 nm by a microplate reader. The concentration of ETHE1 was measured by calculation from a standard curve. The limit of detection for ETHE1 was 7.8 pg/ml.

### Uranium analysis

After different doses of DU injection, uranium content in the kidney was measured by inductively coupled plasma mass spectrometry (ICP-MS, Thermo Finnigan MAT, Bremen, Germany). The methods were as previously described^17^. The quantitation limit for the instrument was 0.002 ppb. Values are expressed as ng/g tissue.

### Western blot analysis

Western blot analysis was used to determine protein levels, as described previously^39^. Equal amounts of samples were electrophoresed on 12% polyacrylamide gels and were then blotted to a poly-vinylidine difluoride membrane. Subsequently the membrane was blocked and then incubated overnight at 4 °C with polyclonal antibody (Abcam, Cambridge, MA, USA) and anti-β-actin polyclonal antibodies (Beyotime) used as the internal reference antibody. The membrane was visualized by enhanced chemiluminescence system (ECL, Millipore, Saint-Quentin-en-Yvelynes, France). Band densities were quantified using the LAS3000 apparatus (Fujifilm, Raytest, Courbevoie, France), and normalized to the total amount of β-actin.

### Cell culture and transient siRNA-mediated knockdown experiments

Human embryonic kidney cell line HEK293 was purchased from the American Type Culture Collection (ATCC, Manassas, VA, USA). Cells were cultured in DMEM medium supplemented with 10% fetal bovine serum and 1% penicillin/streptomycin in a humidified atmosphere with 5% CO_2_ at 37 °C. This medium was replenished every 2 days.

To knock down ETHE1 expression, siRNA targeting ETHE1 was transfected into HEK293 cells using Lipofectamine 2000 (Invitrogen, Carlsbad, CA, USA) according to the manufacturer’s instructions. The design of the specific siRNA sequences and the experimental methods employed for these experiments were described previously^30^. A specific siRNA and negative control siRNA were synthesized by GenePharma (Shanghai, China). The specific sequence was 5′-CAGGCTGACTTACACATTG-3′. A negative control group was set up using a non-specific sequence. Transfection was performed when the cells reached 90% confluence. One hour before transfection, cells were inoculated into 24-well culture plates at a density of 4 × 10^5^ cells/well. Culture medium was used to dilute 2 μl of Oligofectamine, followed by incubation at room temperature for 5 min. After dilution, Oligofectamine was mixed and then incubated with the DNA solution as soon as possible. Cells were incubated in a 37 °C incubator under 5% CO_2_ for appropriate culture times. The expression of ETHE1 was then detected using western blotting.

### Observation of the subcellular localization of ETHE1

HEK293 cells in logarithmic phase were inoculated into specific culture plates for laser scanning confocal microscopy at 2 × 10^5^ cells/plate. After being conventionally cultured for 24 h, the cells were stained with DU (0, 125, 250, and 500 μM) for 24 h. The cells were fixed in 95% ethanol for 30 min. Next, the ethanol was removed, and the cells were washed twice with PBS. Mito-Tracker Green (Life Technologies Corporation; CA, USA) working solution was subsequently added at a final concentration of 50 nM, followed by incubation at 37 °C for 30 min. The Mito-Tracker Green working solution was then discarded, and the cells were washed twice with PBS. The specific rabbit primary antibody diluted 1:200 (anti-ETHE1, Abcam) was subsequently added, followed by incubation at 37 °C for 60 min. After the cells had been washed twice with PBS, Cy3-labelled goat anti-rabbit IgG (H+L) diluted 1:500 was added, followed by incubation at 37 °C for 30 min. The cells’ nuclei were labelled with Hoechst 33342 (Life Technologies Corporation; CA, USA). Finally, after cells had been washed with PBS 3 times, the subcellular localization of ETHE1 was observed under a laser scanning confocal microscope (Leica TCS-SP5, Bensheim, Germany).

### Cell viability measurement

Cell activity was monitored using a cell counting kit-8 (CCK-8) (Dojindo, Shanghai, China) according to the manufacturer’s instruction as our previously described^39^. HEK293 cells were pretreated with or without exogenous ETHE1 (CUSABIO) (100 ng/ml) or ACY-3 (DAKEWE, Bejing, China) (100 ng/ml) for 0.5 h and then incubated in the absence or presence of DU (500 μM) for 24 h. The CCK-8 solution (10 μL) was added to each well (100 μL) followed by incubation for 1 h. The absorbance was measured at 450 nm by a microplate reader (BioRad). The cell viability is expressed as the percentage relative to the control cultures. In addition, a Calcein-AM and PI double staining kit (Dojindo) was used for the double staining and analysis of the living cell and dead cell levels as our previously described^39^. The living cells (yellow-green fluorescence) and dead cells (red fluorescence) were measured by a fluorescence microscope (IX51, Olympus, Tokyo, Japan) with a 490 nm excitation filter.

To further clarify the role of ETHE1 in MT protection from DU, cells transfected with or without ETHE1-specific siRNA or control siRNA were pretreated with or without exogenous MT (Sigma-Aldrich, Santa Clara, CA, USA) (100 nM) for 24 h and then incubated in the absence or presence of DU (500 μM) for 24 h. Cell viability was also determined by a CCK-8 assay.

### Apoptosis detection

Apoptosis was detected in accordance with our previous report^24^. HEK293 cells transfected with or without ETHE1-specific siRNA or control siRNA were incubated in the absence or presence of DU (500 μM) for 24 h. Then cells (approximately 5 × 10^5^/tube) were washed in cold phosphate buffer solution (PBS) and stained with annexin V-fluorescein isothiocyanate (FITC) and propidium iodide (PI) (Dojindo) according to the manufacturer’s protocol. Data were acquired by a FACSCalibur (BD Biosciences Pharmingen, East Rutherford, NJ, USA) and analysed using FlowJo 7.6.4 software (Tree Star Inc., Ashland, OR, USA). In addition, a Calcein-AM and PI double staining kit (Dojindo) was also used for analysis of the living cell and dead cell levels as above.

### Measurement of mitochondrial membrane potential

HEK293 cells were pretreated with or without ETHE1 (100 ng/ml) for 0.5 h and then incubated in the absence or presence of DU (500 μM) for 24 h. Then the cells were used for measurement of mitochondrial membrane potential and observation with transmission electron microscopy (TEM). The mitochondrial membrane potential was measured by a mitochondrial membrane potential assay kit (Beyotime) with a JC-1 probe as our previous study^39^. Mitochondrial membrane potential was monitored by a TCS-SP5 confocal laser scanning microscope (Leica). In this study, increased ratios of green and red fluorescence represented mitochondrial depolarization.

### TEM observation

At the end of the exposure period, HEK293 cells were washed in PBS, and collected by centrifugation at 300 × g for 5 min as previously described^19^. The images were observed with a TECNAI 10 microscope (Philips, FEI, Eindhoven, The Netherlands).

### Statistical analysis

All data were analysed using SPSS 13.0 (SPSS Inc., Chicago, IL, USA). Data were expressed as means ± standard deviations (SD), and analysed by a two-way ANOVA for two independent variables and by a one-way ANOVA for one variable among treatments. Least significant difference (LSD) test was used for multiple comparisons. The results were considered significant at *p *< 0.05 (two-sided).

## Additional Information

**How to cite this article**: Hao, Y. *et al*. Differential protein expression in metallothionein protection from depleted uranium-induced nephrotoxicity. *Sci. Rep.* **6**, 38942; doi: 10.1038/srep38942 (2016).

**Publisher's note:** Springer Nature remains neutral with regard to jurisdictional claims in published maps and institutional affiliations.

## Figures and Tables

**Table 1:** Identification and analysis of differentially expressed proteins in kidney of MT−/− and MT+/+ mice after DU exposure.

Spot No.	NCBI Accession No.	Score	Peptide matches	Sequence coverage (%)	PI	Mass	Protein Name
1	gi|20149748	244	32/37	40	6.28	101618	sarcosine dehydrogenase
2	gi|83921618	217	33/47	55	5.83	69364	ezrin
3	gi|6679525	167	16/23	47	6.18	39193	phosphotriesterase-related protein
5	gi|9506589	127	18/29	47	6.15	36889	fructose-1,6-bisphosphatase 1
6	gi|38371755	210	23/33	72	5.64	31361	N(G), N(G)-dimethylarginine dimethylaminohydrolase 1
7	gi|19525729	141	16/30	58	5.60	35186	λ-crystallin homolog
8	gi|31982632	77	11/31	31	5.30	35264	Aminoacylase-3
10	gi|12963539	141	16/35	72	6.78	27721	protein ETHE1
11	gi|7305599	131	9/17	60	5.77	15766	transthyretin
12	gi|7949005	67	5/16	42	9.36	12489	ATP synthase-coupling factor 6
13	gi|254540162	284	30/31	42	6.83	79871	propionyl-CoA carboxylase α chain
14	gi|6753966	145	14/28	38	6.75	37548	glycerol-3-phosphate dehydrogenase [NAD+]
16	gi|226471	172	13/21	56	6.03	15752	Cu/Zn superoxide dismutase

**Table 2:** The relative fold changes of 13 differentially expressed proteins by 2-DE and image analysis.

Spot No.	Protein Name	MT+/+		MT−/−	
		Control	DU	Control	DU
1	sarcosine dehydrogenase	1.00 ± 0.24	0.32 ± 0.15*	1.03 ± 0.44	0.44 ± 0.24^#^
2	ezrin	1.00 ± 0.11	0.40 ± 0.16*	1.22 ± 0.20^@^	0.54 ± 0.26#
3	phosphotriesterase-related protein	1.00 ± 0.06	0.30 ± 0.03*	0.63 ± 0.22^@^	0.33 ± 0.06#
5	fructose-1,6-bisphosphatase 1	1.00 ± 0.12	0.45 ± 0.11*	0.68 ± 0.28^@^	0.40 ± 0.05#
6	N(G), N(G)-dimethylarginine dimethylaminohydrolase 1	1.00 ± 0.23	0.49 ± 0.04*	1.07 ± 0.34	0.54 ± 0.12#
7	λ-crystallin homolog	1.00 ± 0.05	2.81 ± 0.99*	0.88 ± 0.04^@^	1.81 ± 0.14^#@^
8	Aminoacylase-3	1.00 ± 0.25	0.23 ± 0.05*	1.16 ± 0.37	0.12 ± 0.03^#@^
10	protein ETHE1	1.00 ± 0.32	0.41 ± 0.12*	1.24 ± 0.56	0.20 ± 0.07^#@^
11	transthyretin	1.00 ± 0.16	2.90 ± 1.23*	1.11 ± 0.12	2.55 ± 0.15#
12	ATP synthase-coupling factor 6	1.00 ± 0.18	5.78 ± 1.81*	1.38 ± 0.24	3.91 ± 0.66^#@^
13	propionyl-CoA carboxylase α chain	1.00 ± 0.12	0.45 ± 0.12*	0.69 ± 0.22^@^	0.41 ± 0.05#
14	glycerol-3-phosphate dehydrogenase [NAD+]	1.00 ± 0.22	0.42 ± 0.07*	0.74 ± 0.22^@^	0.42 ± 0.03#
16	Cu/Zn superoxide dismutase	1.00 ± 0.14	0.63 ± 0.23*	0.92 ± 0.37	0.36 ± 0.11^#@^

Values are expressed as the mean ± SD (n = 5). Statistical analysis was performed by a two-way ANOVA and LSD test for multiple comparisons. Spot densities were quantified using Image Master 2D Elite 3.01 software, and normalized to the amount of the control group of MT+/+ mice. *p < 0.05, compared with the control group of MT+/+ mice; ^#^*p* < 0.05, compared with the control group of MT−/− mice; ^@^*p* < 0.05, compared with corresponding MT+/+ mice.
